# Treatment with the immunomodulator FTY720 does not promote spontaneous bacterial infections after experimental stroke in mice

**DOI:** 10.1186/2040-7378-3-2

**Published:** 2011-03-09

**Authors:** Waltraud Pfeilschifter, Bożena Czech-Zechmeister, Marian Sujak, Christian Foerch, Thomas A Wichelhaus, Josef Pfeilschifter

**Affiliations:** 1 Department of Neurology, University Hospital, Goethe University Frankfurt am Main, Germany; 2 Department of General Pharmacology and Toxicology, University Hospital, Goethe University Frankfurt am Main, Germany; 3 Institute of Medical Microbiology and Infection Control, University Hospital, Goethe University Frankfurt am Main, Germany

## Abstract

**Background:**

FTY720, an immunomodulator derived from a fungal metabolite which reduces circulating lymphocyte counts by increasing the homing of lymphocytes to the lymph nodes has recently gained interest in stroke research. The aim of this study was to evaluate the protective efficacy of FTY720 in cerebral ischemia in two different application paradigms and to gather first data on the effect of FTY720 on the rate of spontaneous bacterial infections in experimental stroke.

**Methods:**

Middle cerebral artery occlusion (MCAO) in C57BL/6 mice (strain J, groups of 10 animals) was performed with two different durations of ischemia (90 min and 3 h) and FTY720 was applied 2 h after vessel occlusion to study the impact of reperfusion on the protective potency of FTY720. Lesion size was determined by TTC staining. Mice treated with FTY720 or vehicle were sacrificed 48 h after 90 min MCAO to determine the bacterial burden in lung and blood.

**Results:**

FTY720 1 mg/kg significantly reduced ischemic lesion size when administered 2 h after the onset of MCAO for 3 h (45.4 ± 22.7 mm^3 ^vs. 84.7 ± 23.6 mm^3 ^in control mice, p = 0.001) and also when administered after reperfusion, 2 h after the onset of MCAO for 90 min (31.1 ± 28.49 mm^3 ^vs. 69.6 ± 27.2 mm^3 ^in control mice, p = 0.013). Bacterial burden of lung homogenates 48 h after stroke did not increase in the group treated with the immunomodulator FTY720 while there was no spontaneous bacteremia 48 h after MCAO in treated and untreated animals.

**Conclusions:**

Our results corroborate the experimental evidence of the protective effect of FTY720 seen in different rodent stroke models. Interestingly, we found no increase in bacterial lung infections even though FTY720 strongly reduces the number of circulating leukocytes.

## Background

Despite decades of basic and translational research, there is still no pharmaceutical stroke treatment besides thrombolysis which has been proven to be effective in humans [1]. To promote the transition of scientific evidence from animal studies on experimental stroke, the Stroke Therapy Academic Industry Roundtable (STAIR) has formulated a set of criteria for the conduct, reporting, and analysis of animal data which include the points that drug candidates should be tried by different research teams, in different stroke models, in different animal species, and at different time points [2]. One drug, which has recently gained a lot of interest and already fulfils some of these criteria on the basis of the current experimental evidence is the sphingosine 1-phosphate (S1P) analogue and immunomodulator FTY720 (fingolimod).

FTY720 is phosphorylated to yield the biologically active substance FTY720-phosphate by the ubiquitously available sphingosine kinase-2 and to a lesser extent by sphingosine kinase-1 [3]. FTY720-phosphate can activate four of the five G protein-coupled S1P receptors known so far [4]. It leads to a downregulation of autoimmune-inflammatory responses by inducing the internalization of the S1P_1 _receptor of lymphocytes and thus inhibits the lymphocyte egress from the lymph node into the systemic circulation [4], while the functional responses of the lymphocytes remain relatively unaltered [5].

FTY720 has been shown to reduce lesion size and improve neurological outcome after experimental stroke in mice [6,7] and rats [7,8] with a therapeutic time window of up to four hours after the induction of ischemia [7]. It has been shown to reduce brain damage after stroke in models of transient [6-8] and permanent [7] middle cerebral artery occlusion (MCAO) by reducing the infiltration of neutrophils into the ischemic lesion [6], attenuating the activation of microglia/macrophages [6], reducing hallmarks of apoptotic cell death within the lesion and activating survival pathways via Akt and ERK phosphorylation [8] in the ischemic brain. The protective effect on lesion size was still present at 72 h after MCAO [8] and FTY720-treated mice performed better than controls in a behavioural test performed 15 days after experimental stroke [7].

It is well known that cerebral ischemia has a profound effect on the immune system, leading to an immunosuppression with reduced leukocyte counts and reactivity as well as an atrophy of secondary lympoid tissues after stroke [9]. Stroke patients are prone to infections, predominantly chest and urinary tract infections [10] and pneumonia is the complication with the highest attributable risk of death in the acute phase of stroke [11]. In the MCAO model of cerebral ischemia, mice after stroke showed higher rates of spontaneous bacterial infections than control animals [12] and were more susceptible to infection after nasal inoculation of *S. pneumoniae *[13]. In this context, it is of great interest whether FTY720 as an immunomodulatory substance which reduces the number of circulating leukocytes, especially T cells, is associated with a higher rate of infectious complications. The aim of this study was to evaluate the efficacy of FTY720 in two different application paradigms before and after vessel recanalization in large territorial infarctions and to gather first data on the effect of FTY720 on the rate of spontaneous bacterial infections in experimental stroke.

## Methods

### Animals and sample size calculation

Male C57BL/6 mice (10 weeks old, strain J) were used in accordance with the National Institute of Health Guide for the Care and Use of Laboratory Animals (NIH Publications No. 80-23, revised 1996). The experiments were approved by the local governmental authorities (Regierungspraesidium Darmstadt, Germany, approval number F143/34). In pilot experiments of the two application paradigms for FTY720 tested in this study, we found a reduction in lesion size of approximately 50% with a standard deviation of 0.33 to 0.5 of the lesion size leading to an effect size (Cohen's d) of 1.697 [13]. To detect this effect size with an alpha level of 0.05 and a statistical power of 0.9, nine animals per group were needed. 10 animals per group were randomized to receive either FTY720 or vehicle.

### Middle cerebral artery occlusion

The operator was blinded to the treatment status of the animals. Transient MCAO was performed as described previously [6] in groups of 10 vs. 10 animals per application paradigm. Mice were anaesthetized with 1.5% isoflurane (Forene; Abbott, Wiesbaden, Germany) and 0.1 mg/kg buprenorphine (Temgesic; Essex Pharma, Munich, Germany) under spontaneous respiration. Focal cerebral ischemia was induced by introducing a silicone-coated 7-0 monofilament until it occluded the ostium of the right MCA. Regional cerebral blood flow was monitored by laser Doppler flowmetry (PF5010, Perimed, Järfälla, Sweden) to confirm vessel occlusion. The filament was withdrawn after the indicated time points, i.e. 3 h or 90 min, to allow reperfusion of the ischemic hemisphere. Animals were sacrificed at 24 h, 36 h or 48 h after assessment of their global neurological functions with a 5-point neuroscore (0 = no deficit, 1 = failure to extend left paw, 2 = circling to the left, 3 = falling to the left, 4 = unable to walk spontaneously, 5 = death). All animals that showed a drop of the Doppler flow below 40% of the initial value and a neurological deficit (neuroscore ≥ 1) were included in the analysis.

### Dosing and administration of FTY720

FTY720 (Cayman Chemicals Europe, Tallinn, Estonia) was dissolved in 0.9% NaCl at a final concentration of 125 μg/ml yielding a clear liquid and 200 - 250 μl were injected intraperitoneally 2 h after the onset of cerebral ischemia. Control animals received the corresponding volume of 0.9% NaCl i.p. Syringes were prepared by an allocator and administered by the operator in a blinded fashion.

### Assessment of ischemic lesion volume

Twenty-four hours after the onset of ischemia, brains were removed and cut into sections of 1 mm thickness using a mouse brain matrix (RBM 2000C; ASI Instruments, Warren, Mich; USA). The brain sections were stained in 2% (w/v) 2,3,5-triphenyltetrazolium chloride (TTC, Merck, Darmstadt, Germany) in phosphate buffer at 37°C for 10 minutes. Brain slices were digitized and infarct volumes were measured by an observer who was blinded to the treatment conditions with the National Institutes of Health Image J software analysing four slices of identical positions adjacent to the bregma. The correction for edema was established by multiplying the infarct section volume by the ratio of the contralateral to the ischemic hemisphere section volume.

### Microbiological Analyses

Mice were subjected to 90 min MCAO and substance application at the onset of reperfusion. Thereafter, mice were returned to their cages for 24 h, 36 h and 48 h to observe whether they developed spontaneous bacterial infections. Only mice that showed a neurological deficit after 24 h were used for further analyses. 24 h, 36 h and 48 h after the induction of cerebral ischemia, the anesthetized mice were washed in 70% alcohol under sterile conditions. Lungs were removed and homogenized in sterile phosphate-buffered solution. For the determination of the bacterial load, Blood was drawn from the heart into an EDTA collection tube and 100 μl were serially diluted and plated onto blood and chocolate agar for anaerobic and aerobic incubation, respectively. 100 μl of lung homogenate were serially diluted and plated onto appropriate agar as described above. Colonies were counted after incubation for 48 h at 37°C. Lung infection was defined as any bacterial growth from the sterilely collected lung samples. Status of lung infection was categorized into four groups (no infection, <5.000 CFU/ml, >50.000 CFU/ml and death). Accordingly, these groups were further dichotomized into "moderate or no infection" and "severe infection or death".

### Statistical analysis

Graph Pad Prism 4 (Graph Pad Software Inc., La Jolla, CA, USA) was used for statistical analysis. Results are given as mean ± SD and graphically presented as a box and whiskers plot depicting the mean, extreme values and the 25 - 75 interquartile range. Statistical significance was assessed with an unpaired, two-tailed student's t-test and a Mann-Whitney-Test where indicated. Statistical significance between the dichotomized infection rates in FTY- and vehicle-treated animals was tested with a two-sided chi-square test.

## Results

### FTY720 significantly reduces lesion size in large territorial infarctions

**Figure 1:**
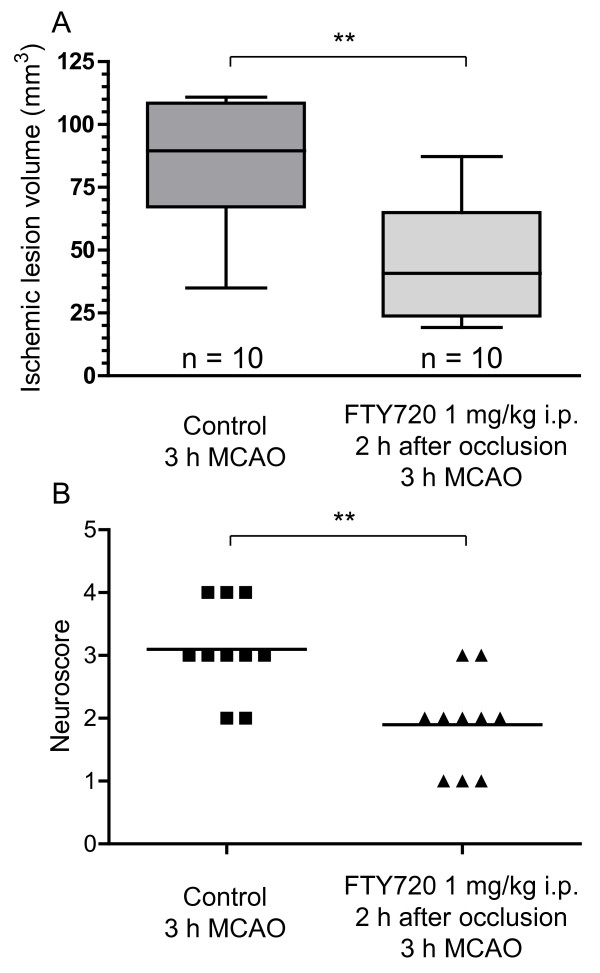
**A: FTY720 reduces ischemic lesion size in large territorial infarctions**. MCAO was performed for 3 h. FTY720 or vehicle were administered 2 h after the induction of cerebral ischemia (n = 10 in both groups). Data are depicted as box and whiskers plots showing the 25 to 75 percentile and the extreme values. Statistical significance was assessed with students unpaired t-test. ** p < 0.01. B: The functional neurological status of the mice was tested with a 5-point neuroscore after 24 h. Medians are given and statistical significance was tested with a Mann-Whitney test. ** p < 0.01.

Application of FTY720 (1 mg/kg i.p.) 2 h after the onset of cerebral ischemia significantly reduced ischemic lesion size in mice subjected to three hours MCAO (45.4 ± 22.7 mm^3 ^vs. 84.7 ± 23.6 mm^3 ^in control mice, p = 0.001, n = 10 vs. 10, Figure 1A). Functionally, this was reflected by a less severe neurological deficit of the FTY720-treated animals (mean neuroscore value 2, range 1 - 3 vs. mean 3, range 2 - 4 in control mice, p = 0.005, Figure 1B). None of the animals died during the operation and during the 24 h observation time.

### FTY720 is also protective when administered after reperfusion

**Figure 2:**
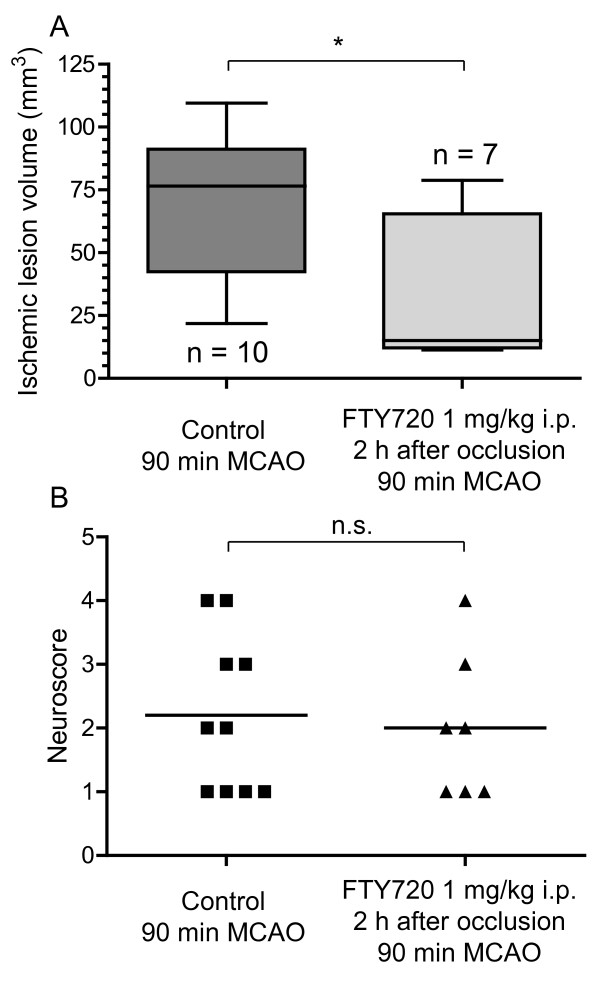
**A: FTY720 reduces ischemic lesion size when administered after reperfusion**. MCAO was performed for 90 min and FTY720 (n = 10) or vehicle (n = 7) were applied 2 h after vessel occlusion. Data are depicted as box and whiskers plots showing the 25 to 75 percentile and the extreme values. Statistical significance was assessed with student's unpaired, two-tailed t-test. * p < 0.05. B: The functional neurological status of the mice was tested with a 5-point neuroscore after 24 h. Medians are given and statistical significance was tested with a Mann-Whitney test. n.s. = not significant.

In a second experimental paradigm, we administered FTY720 (1 mg/kg i.p.) 2 h after the onset of MCAO for 90 minutes, hence after the induction of reperfusion. Even under these conditions, FTY720 significantly halved the ischemic lesion size at 24 h (31.1 ± 28.5 mm^3 ^vs. 69.6 ± 27.2 mm^3 ^in control mice, p = 0.013, n = 7 vs. 10, Figure 2A), but there was only a tendency towards a better functional outcome in FTY720-treated animals (mean neuroscore value 2, range 1-4 vs. mean 2, range 1-4 in control animals, p = 0.81, Figure 2B). Three mice of the FTY720-treated group were excluded (1 did not show a neurological deficit, 1 developed SAH and 1 died during the induction of anesthesia).

### FTY720 does not increase the rate of bacterial infections at48 h after MCAO

**Figure 3:**
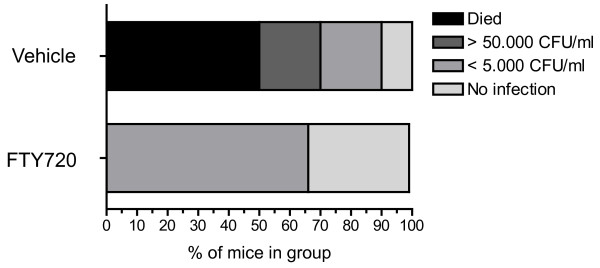
**FTY720 does not lead to an increase in bacterial lung infections after stroke**. Mice received FTY720 1 mg/kg (n = 3) or vehicle (n = 10) 2 h after the onset of cerebral ischemia and lung samples were taken for microbiological analysis. Bacterial colonies were counted after incubating 48 h at 37°C. The degree of infection was categorized into four groups and the percental number of mice per group is depicted.

We randomized 3 vs. 3 animals to vehicle or FTY720 treatment. In untreated animals, there was an excess death rate, which prompted us to include more animals in this group *a posteriori *(10 mice in total) to obtain a reasonable number for microbiological analysis. All of the 5 animals that died in the control group did so between the 36 h and the 48 h observation time points. But also in the surviving 5 animals, the rate of severe lung infections >50.000 CFU/ml was considerably high while none of the FTY720-treated animals died during the observation period or showed a severe infection (Figure 3). Dichotomized into "moderate or no infection" and "severe infection or death", we found significantly less severe pulmonary infections in the FTY720-treated compared to the vehicle-treated group (Table 1, p = 0.033). We did not detect bacteremia in any of the mice (data not shown) and we did not detect lung infections in mice whose lungs were sampled after shorter time spans (24 h and 36 h, data not shown).

**Table 1:** Pulmonary infections 48 h after 90 min MCAO

	Severe infection or death	Moderate or no infection	Sum
Vehicle	7	3	10

FTY720 1 mg/kg	0	3	3

Sum	7	6	13

Contingency table showing the number of mice in each group after the degree of infection was dichotomized into "moderate or nor infection" and "severe infection or death". Statistical significance was assayed with a Chi-square test, p = 0.033.

## Discussion

Adding to the considerable body of experimental evidence on the protective effect of FTY720 in stroke [6-8], our experiments show that FTY720 is also protective in large hemispheric infarctions within a therapeutic time window that is achievable in patient care. Even if administered after the critical time point of reperfusion, it still leads to a significant reduction in ischemic lesion size.

After two successful phase III trials [14,15] FTY720 has been approved as the first orally available drug to treat relapsing-remitting multiple sclerosis by the FDA in 2010. In over 1200 patients, FTY720 was generally well tolerated and did not lead to a significantly elevated rate of infections, but two cases of severe viral infections occurred in the verum groups. The highest dose in the aforementioned trials was 1.25 mg (approx. 0.02 mg/kg) administered as a chronic daily dose, which is fiftyfold lower than the dose of 1 mg/kg required for a protective effect in experimental stroke in mice [7]. The highest dose reported in humans so far was 5 mg administered on a daily basis [16] and dosing was limited by the S1P receptor-mediated bradycardia which occurs within hours after FTY720 intake.

FTY720 leads to a reduction of circulating immune cells, especially T cells, as we have shown previously [6]. At 1 mg/kg, the number of circulating neutrophils is also reduced [[17], and Pfeilschifter W. unpublished observations]. Especially in consideration of the well-described phenomenon of stroke-induced immunosuppression (cerebral injury induced immune deficiency syndrome, CIDS) [18], it is important to take account of the influence of FTY720-treatment on the rate of stroke-associated infections because they constitute an important factor that modulates the outcome of stroke patients [11]. We observed varying degrees of bacterial colonization in the lungs of mice 48 h after stroke and only very few lung homogenate samples remained sterile during incubation. This is in accordance with an earlier study [12], corroborating the hypothesis that mice after stroke are prone to bacterial lung infections. However, in contrast to the aforementioned study, which documented the first cases of bacterial lung infection after 12 h with a constant rise of the incidence of infection and bacterial load over the following 72 h as well as blood stream infections beginning at 24 h after stroke, we neither found bacteremia at all after 48 h nor lung infections at the 24 h and 36 h time points. There are several possible explanations for this divergence. Since both experiments detected spontaneous bacterial infections, the local spectrum of bacteria in the respective animal care facilities might be one determinant. Since the degree of CIDS is critically determined by the size of the brain lesion both in humans [19] and in mice [20], differences in ischemic lesion size might also explain differences in the susceptibility to bacterial infections. An analysis of the results of our microbiological examination dichotomized in moderate and severe pulmonary infections even showed a significantly lower rate of severe infections in FTY720-treated animals compared to controls. One possible explanation is that given the immunosuppressive effect of acute brain lesions, the reduction of lesion size achieved by FTY720 might outweigh its immunomodulatory effect. Nonetheless, given the small number of animals, these results have to be interpreted with utmost care.

## Conclusions

While corroborating the protective effect of FTY720 in stroke in various rodent models and modes of application, our data also suggest that FTY720 does not further aggravate stroke-induced immune depression to a clinically relevant degree as evaluated microbiologically.

## Competing interests

The authors declare that they have no competing interests.

## Authors' contributions

WP, BC and TAW conceived the experiments, BC (blinded operator) and MS (allocator) conducted the experiments, WP (blinded lesion size analysis), BC, MS and TAW analyzed the data, WP drafted the manuscript, TAW, CF and JP critically revised the manuscript. All authors read and approved the final manuscript.
